# Artesunate inhibits hepatocellular carcinoma cell migration and invasion through OGA-mediated *O*-GlcNAcylation of ZEB1

**DOI:** 10.1515/biol-2025-1109

**Published:** 2025-08-05

**Authors:** Ying Li, Yuan Zhang, Wei Lu, Yun Chen, Xiaoling Qian

**Affiliations:** Special Inspection Department, Hangzhou TCM Hospital affiliated to Zhejiang Chinese Medical University, Hangzhou, 310007, China; Dept Intense Care Unit, Hangzhou TCM Hospital affiliated to Zhejiang Chinese Medical University, No. 453, Stadium Road, Hangzhou, Zhejiang, 310007, China

**Keywords:** hepatocellular carcinoma, artesunate, OGA, *O*-GlcNAcylation, ZEB1, migration, invasion

## Abstract

Metastasis remains a major challenge to improve the survival of patients with hepatocellular carcinoma (HCC). Artesunate is an antimalarial drug that also has anti-cancer properties. Additionally, *O*-GlcNAcylation has been implicated in cancer progression. In this study, we investigated whether artesunate regulated HCC cell migration and invasion and explored its impact on protein *O*-GlcNAcylation. Cellular functions, including viability, migration, and invasion, were evaluated using the cell counting kit-8, scratch assay, and Transwell analysis. Molecular docking and biolayer interferometry were employed to assess the binding interaction between artesunate and OGA. Furthermore, the *O*-GlcNAcylation of ZEB1 was examined using immunoprecipitation, cycloheximide chase assay, and immunoblotting. Our results demonstrated that artesunate significantly inhibited HCC cell viability, migration, and invasion. OGA expression was increased in HCC cells after artesunate treatment. Artesunate directly bound to OGA, and OGA knockdown reversed the inhibition of malignant behaviors induced by artesunate. Additionally, OGA suppressed the *O*-GlcNAcylation of ZEB1 at the Ser670 site, decreasing protein stability. Knockdown of ZEB1 inhibited HCC cellular behaviors. In conclusion, artesunate inhibits HCC cell migration and invasion by binding to OGA, which removes the *O*-GlcNAcylation of ZEB1 at the Ser670 site. These findings provide a new action mechanism for artesunate to treat HCC.

## Graphical abstract

Artesunate combines with OGA, which promotes *O*-GlcNAcylation of ZEB1 and thus reduces the protein stability of ZEB1, further inhibiting migration and invasion of hepatocellular carcinoma cells.

**Figure:**
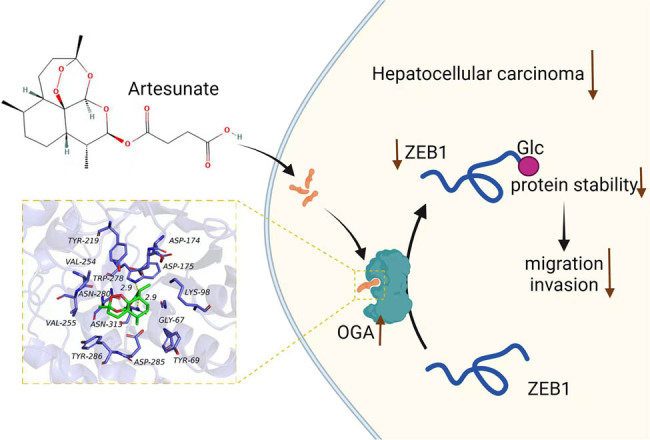

## 1 Introduction

Hepatocellular carcinoma (HCC) is the fourth leading cause of cancer-related mortality worldwide [1]. In addition to genetic predisposition, major risk factors for HCC include hepatitis virus infection, cirrhosis, and non-alcoholic fatty liver disease [2,3]. Early diagnosis plays a critical role in the treatment and prognosis of HCC, as it enables the detection of tumors at a curable stage [4]. However, the majority of patients are diagnosed at advanced stages of the disease. Distant metastasis is a key factor contributing to the poor survival rates observed in advanced HCC patients. Current therapeutic strategies, such as hepatectomy, liver transplantation, and thermal ablation, exhibit limited efficacy in these cases [5]. Furthermore, HCC recurrence rates remain alarmingly high, with up to 70% of patients experiencing recurrence within 5 years post-treatment [6]. Sorafenib remains the only approved systemic therapy for advanced HCC. While targeted therapies and immunotherapies have shown promise in improving survival rates, their clinical efficacy remains inadequately supported by robust evidence [7]. Therefore, a deeper understanding of the molecular mechanisms underlying HCC metastasis is essential to develop novel and effective therapeutic interventions.

Artesunate, a sesquiterpene lactone derivative of artemisinin, has been chemically modified to enhance solubility, absorption, and pharmacokinetic properties, thereby overcoming artemisinin resistance. It is widely used for the treatment of moderate to severe malaria [8,9]. Beyond its antimalarial properties, artesunate exhibits a broad spectrum of biological activities, including antiviral, anti-inflammatory, antitumor, and immunomodulatory effects [10]. Recent studies have highlighted its potential therapeutic applications in respiratory diseases, autoimmune disorders, COVID-19, and various malignancies [11,12,13,14]. Specifically, artesunate has been shown to enhance the sensitivity of HCC cells to sorafenib [15,16] and significantly reduce tumor burden in HCC models [17]. However, the precise molecular mechanisms by which artesunate exerts its antitumor effects in HCC remain poorly understood.

*O*-GlcNAcylation (*O*-linked β-*N*-acetylglucosaminylation) is a dynamic and reversible post-translational modification that plays a critical role in regulating various pathophysiological processes. This modification is catalyzed by *O*-GlcNAc transferase (OGT), which adds *O*-linked *N*-acetylglucosamine to serine and threonine residues of target proteins, while *O*-GlcNAcase (OGA) removes this modification [18]. *O*-GlcNAcylation is essential for modulating enzyme activity, protein localization, protein–protein interactions, and protein degradation. Dysregulation of *O*-GlcNAcylation has been implicated in the development and progression of numerous diseases, particularly cancers [19,20]. In HCC, *O*-GlcNAcylation has been shown to influence tumor growth, metastasis, stemness, and drug resistance [21]. Therefore, a deeper understanding of the regulatory mechanisms of *O*-GlcNAcylation in HCC is crucial for elucidating the molecular basis of HCC pathogenesis.

In this study, we explored the effects of artesunate on HCC cell migration and invasion and investigated the underlying molecular mechanisms through *in vitro* experiments. Our findings demonstrate that artesunate significantly inhibits these malignant phenotypes. Furthermore, we provide evidence that artesunate exerts its antitumor effects by modulating OGA-mediated *O*-GlcNAcylation of ZEB1, a key transcription factor involved in cancer progression.

## 2 Materials and methods

### 2.1 Cell culture

The human liver epithelial cell line (THLE3) and one HCC cell line (Hep3B) were obtained from the American Type Culture Collection (ATCC, Manassas, VA, USA). An additional HCC cell line, HCCLM3, was procured from the Delf Cell Bank (hfwanwu, Hefei, China). THLE3 cells were cultured in Roswell Park Memorial Institute-1640 medium (GIBCO BRL, Grand Island, NY, USA) supplemented with 10% fetal bovine serum (FBS; GIBCO BRL) and maintained at 37°C in a humidified atmosphere containing 5% CO_2_. HCC cells (Hep3B and HCCLM3) were cultured in Dulbecco’s Modified Eagle’s Medium (GIBCO BRL) supplemented with 10% FBS under the same conditions (37°C, 5% CO_2_).

### 2.2 Determination of cell viability

**Figure 1:**
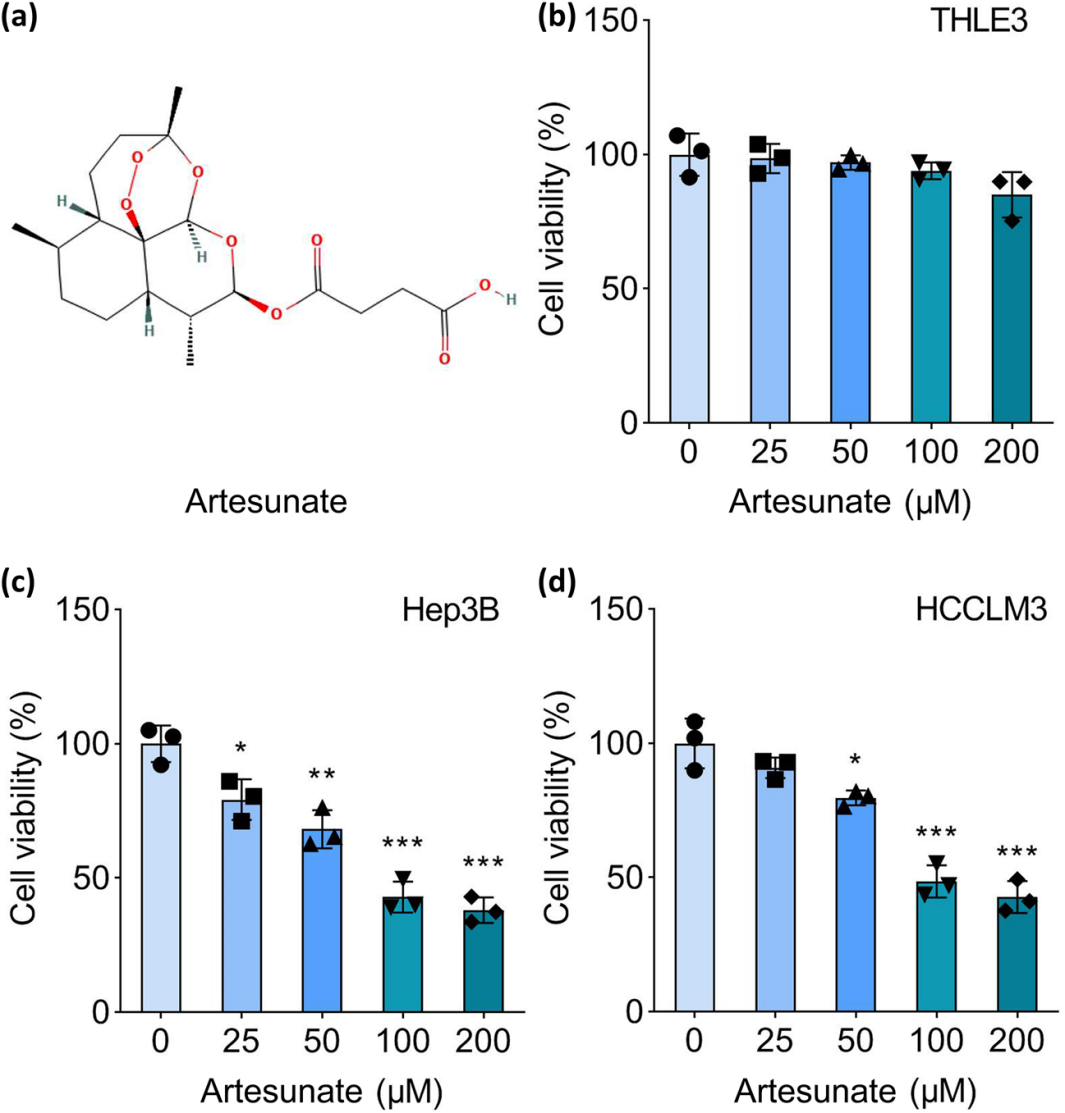
Artesunate inhibits HCC cell viability. (a) Chemical structure of artemisinin. (b) Normal THLE3, (c) Hep3B, and (d) HCCLM3 cells were treated with 0, 25, 50, 100, and 200 μM artesunate for 48 h, and cell viability was measured using the cell counting kit-8. *P < 0.05, **P < 0.01, and ***P < 0.001 vs the 0 μM group.

All cells were seeded into 96-well plates at a density of approximately 2,000 cells per well. Following cell attachment, they were treated with a gradient of artesunate concentrations (0, 25, 50, 100, and 200 μM; MedChemExpress, Monmouth Junction, NJ, USA; Figure 1a) for 48 h. After treatment, 10 μL of cell counting kit-8 solution (Dojindo, Kumamoto, Japan) was added to each well, and the cells were incubated for an additional 2 h. The absorbance at 450 nm was measured using a microplate reader to assess cell viability.

### 2.3 Wound healing assay

HCC cells were seeded into 24-well plates and cultured until they reached a confluent monolayer. A uniform wound was created in the monolayer using a sterile 20 μL pipette tip. After washing with phosphate-buffered saline (PBS), the cells were imaged to establish the baseline (0 h). The plates were then incubated at 37°C with 5% CO_2_ for 24 h, after which the cells were imaged again to assess wound closure (24 h).

### 2.4 Transwell assay

Transwell chambers (24-well, pore size: 8 μm; Corning, Corning, NY, USA) were used to assess cell invasion and migration. For invasion assays, the chambers were pre-coated with Matrigel, while uncoated chambers were used for migration assays. A cell suspension containing approximately 1 × 10^5^ cells was added to the upper chamber, and the lower chamber was filled with complete medium. The chambers were incubated at 37°C for 24 h. After incubation, cells that had invaded or migrated through the membrane were fixed, stained with 0.1% crystal violet, and visualized under a light microscope.

### 2.5 Immunoblotting

HCC cells were lysed using radioimmunoprecipitation assay buffer (Beyotime, Shanghai, China). Protein concentrations were quantified, and equal amounts of protein were separated by 10% sodium dodecyl sulfate-polyacrylamide gel electrophoresis and subsequently transferred onto polyvinylidene difluoride membranes (Millipore, Billerica, MA, USA). The membranes were blocked with 5% skim milk for 1 h at room temperature and then incubated overnight at 4°C with primary antibodies, followed by incubation with a horseradish peroxidase (HRP)-conjugated secondary antibody for 1 h at room temperature. Protein bands were visualized using BeyoECL Plus solution (Beyotime).

The primary antibodies used in this study included anti-*O*-GlcNAc/RL2 (ab2739, Abcam, Cambridge, UK), anti-OGA (ab68522, Abcam), anti-OGT (sc-74546, Santa Cruz Biotechnology, Santa Cruz, CA, USA), anti-ZEB1 (ab181451, Abcam), and anti-GAPDH (ab8245, Abcam). The secondary antibody was HRP-conjugated rabbit anti-mouse IgG (ab6728, Abcam).

### 2.6 Biolayer interferometry (BLI)

BLI experiment was performed using the OCTET RED R8 system (Sartorius, Göttingen, Germany) by a standard protocol as previously described [22]. OGA protein was loaded on NTA biosensors using the following conditions: baseline 1,300 s and loading 3,600 s. Different concentrations of artesunate were prepared using PBS containing 5% dimethyl sulfoxide (DMSO). Subsequently, 200 μL of each artesunate solution was transferred to a 96-well black polypropylene plate containing PBS with 5% DMSO. Binding interactions were monitored in real-time using the OCTET RED R8 instrument, and data were analyzed using Octet Data Analysis 10.0 software.

### 2.7 Molecular docking

The molecular structure of artesunate was obtained from the PubChem database and converted into the appropriate format using Chem3D. The crystal structure of OGA was retrieved from the RCSB PDB. The crystal water removed, hydrogen atoms added, and energy minimized were performed using the Protein Preparation Wizard on the Maestro 11.9 platform. Molecular docking was performed using the Schrödinger Maestro software through the Glide module. Standard precision flexible ligand mode was used to dock the protein and ligands. The binding compound was visualized using Pymol2.1 software.

### 2.8 Cell transfection

Hep3B and HCCLM3 cells were seeded into six-well plates and cultured for 24 h prior to transfection. Short hairpin RNA targeting OGA (shOGA), shZEB1, non-targeting control shRNA (shNC), OGA overexpression vector, and empty vector (GenePharma, Shanghai, China) were transfected into HCC cells using Lipofectamine 2000 (Invitrogen, Carlsbad, CA, USA) according to the manufacturer’s instructions. After 6 h of transfection, the medium was replaced with fresh complete medium. Cells were harvested 48 h post-transfection for subsequent analyses.

### 2.9 Quantitative real-time polymerase chain reaction (qRT-PCR)

TRIzol reagent was used to isolate total RNA from cells. RNA concentration was determined using the Nanodrop 8000. Total RNA (about 1 μg) was reverse transcribed to cDNA using the PrimeScript 1st strand cDNA synthesis kit (Takara, Dalian, China). Real-time PCR was carried out using FastStart Universal SYBR Green master mix (Roche, Basel, Switzerland) on the LightCycler 480 instrument (Roche). The Ct value was obtained and the results were calculated using the 2^−ΔΔCt^ method. GAPDH served as the internal control. The primers with the following sequences were used in real-time PCR: OGA sense: 5′-CATAGGATGTTTTGGCGAGAGAT-3′ and anti-sense: 5′-GGTGAGATCGCATAGATGAACTC-3′ and GAPDH sense: 5′-CTGGGCTACACTGAGCACC-3′ and anti-sense: 5′-AAGTGGTCGTTGAGGGCAATG-3′.

### 2.10 Immunoprecipitation

The *O*-GlcNAcylation levels of proteins were assessed using an immunoprecipitation kit (Beyotime, Shanghai, China) followed by immunoblotting. Briefly, Hep3B cells were lysed in lysis buffer supplemented with a protease inhibitor cocktail, and the lysate was clarified by centrifugation at 14,000 × *g* for 5 min. Protein A/G magnetic beads were incubated with 500 μL of antibody working solution (targeting *N*-cadherin, Vimentin, MMP3, MMP9, ZEB1, E-cadherin, and ZO-1) or normal IgG working solution at room temperature for 1 h. The clarified lysate was then incubated with the antibody-beads complex at room temperature for 2 h. After incubation, the magnetic beads were separated and washed three times with lysis buffer. The *O*-GlcNAcylation levels of the immunoprecipitated proteins were analyzed by immunoblotting using an anti-*O*-GlcNAc (RL2) antibody.

### 2.11 Cycloheximide (CHX) chase experiment

To assess the effect of OGA on ZEB1 protein stability, transfected cells were treated with 100 μM CHX for 0, 6, 12, and 24 h. To inhibit OGA activity, cells were treated with 10 μM Thiamet-G (TMG; Sigma-Aldrich, St. Louis, MO, USA). After treatment, cells were harvested, and ZEB1 protein levels were analyzed by immunoblotting. GAPDH was used as an internal loading control for normalization.

### 2.12 *O*-GlcNAcylation site prediction

The DictyOGlyc 1.1 server online database was used to predict possible *O*-GlcNAcylation sites in ZEB1.

### 2.13 Statistical analysis

Data were acquired from three independent repeated experiments and analyzed using the GraphPad Prism 8.0 software. The results were expressed as mean ± standard deviation. Comparisons were performed using Student’s *t*-test (two groups) and one-way analysis of variance (three groups). *P* < 0.05 was considered statistically significant.

## 3 Results

### 3.1 Artesunate inhibits HCC cell viability

To evaluate the effects of artesunate on HCC progression, Hep3B and HCCLM3 cells were treated with increasing concentrations of artesunate. THLE3 cells, a non-tumorigenic liver epithelial cell line, were also treated with artesunate as a control. The results demonstrated that artesunate had no significant impact on the viability of THLE3 cells (Figure 1b). In contrast, artesunate significantly reduced the viability of Hep3B and HCCLM3 cells in a dose-dependent manner (Figure 1c and d). According to the cell viability results, the IC50 value was determined. We found IC50 = 745.0 μM in THLE3 cells, IC50 = 98.5 μM in Hep3B cells, and IC50 = 129.9 μM in HCCLM3 cells (Figure S1). Based on these findings, 100 μM artesunate, the lowest concentration that exerted a pronounced inhibitory effect on cell viability, was selected for subsequent experiments.

### 3.2 Artesunate suppresses migration and invasiveness of HCC cells

**Figure 2:**
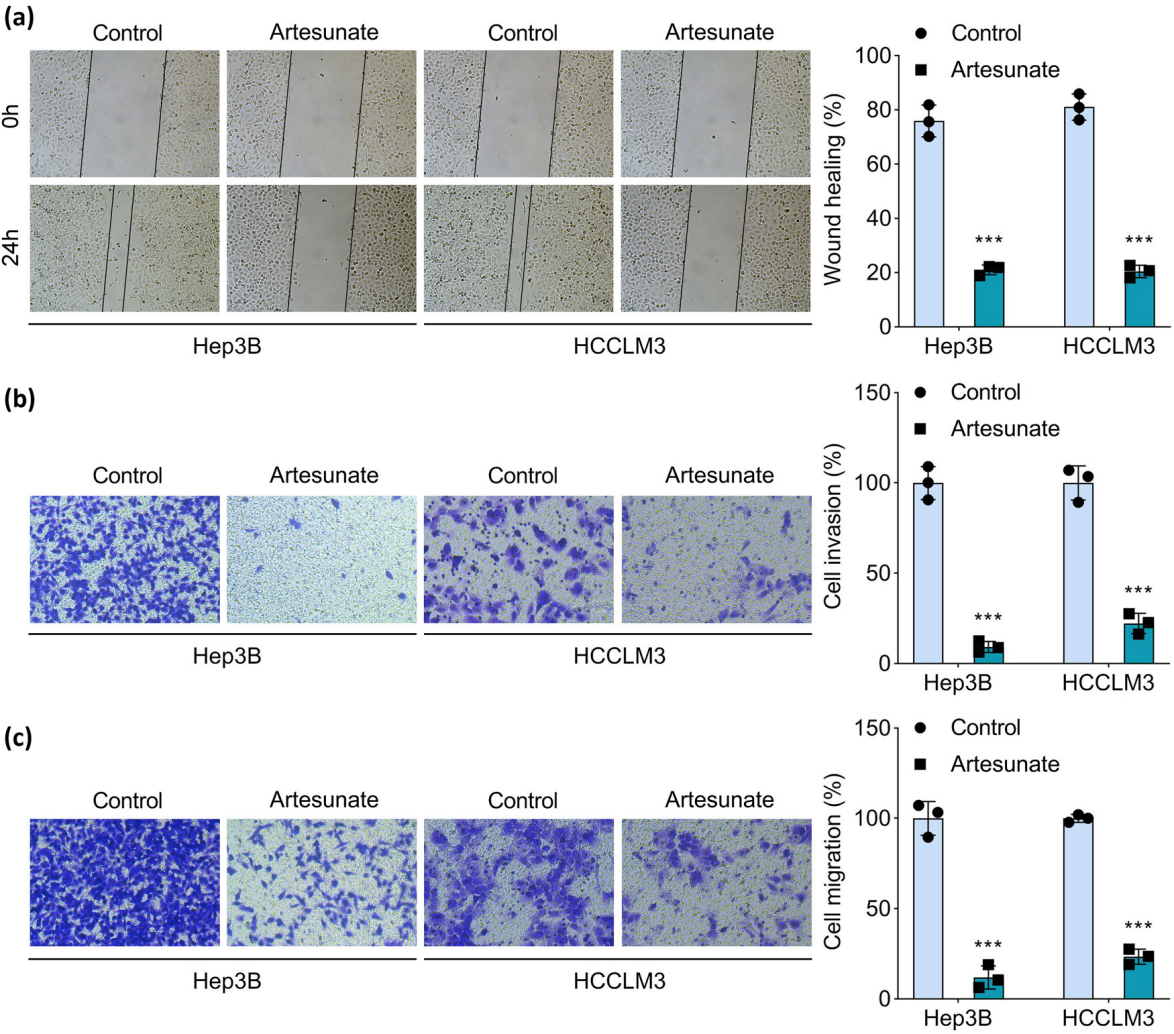
Artesunate suppresses the cell migration and invasiveness of HCC cells. Hep3B and HCCLM3 cells were treated with artemisinin, and (a) cell migration was measured using the wound healing assay; the Transwell assay was conducted to determine (b) cell invasion and (c) migration. ***P < 0.001 vs the control group.

Tumor cell migration and invasion are critical processes underlying tumor metastasis. To investigate the effects of artesunate on these phenotypes, scratch wound healing and Transwell assays were performed. The results revealed that artesunate significantly reduced the percentage of wound closure and the number of migrated cells (Figure 2a and c), indicating its inhibitory effect on HCC cell migration. Furthermore, artesunate treatment markedly decreased the number of invaded Hep3B and HCCLM3 cells (Figure 2b). Collectively, these findings demonstrate that artesunate effectively suppresses both migration and invasion of HCC cells.

### 3.3 Artesunate inhibits OGA-mediated *O*-GlcNAcylation

**Figure 3:**
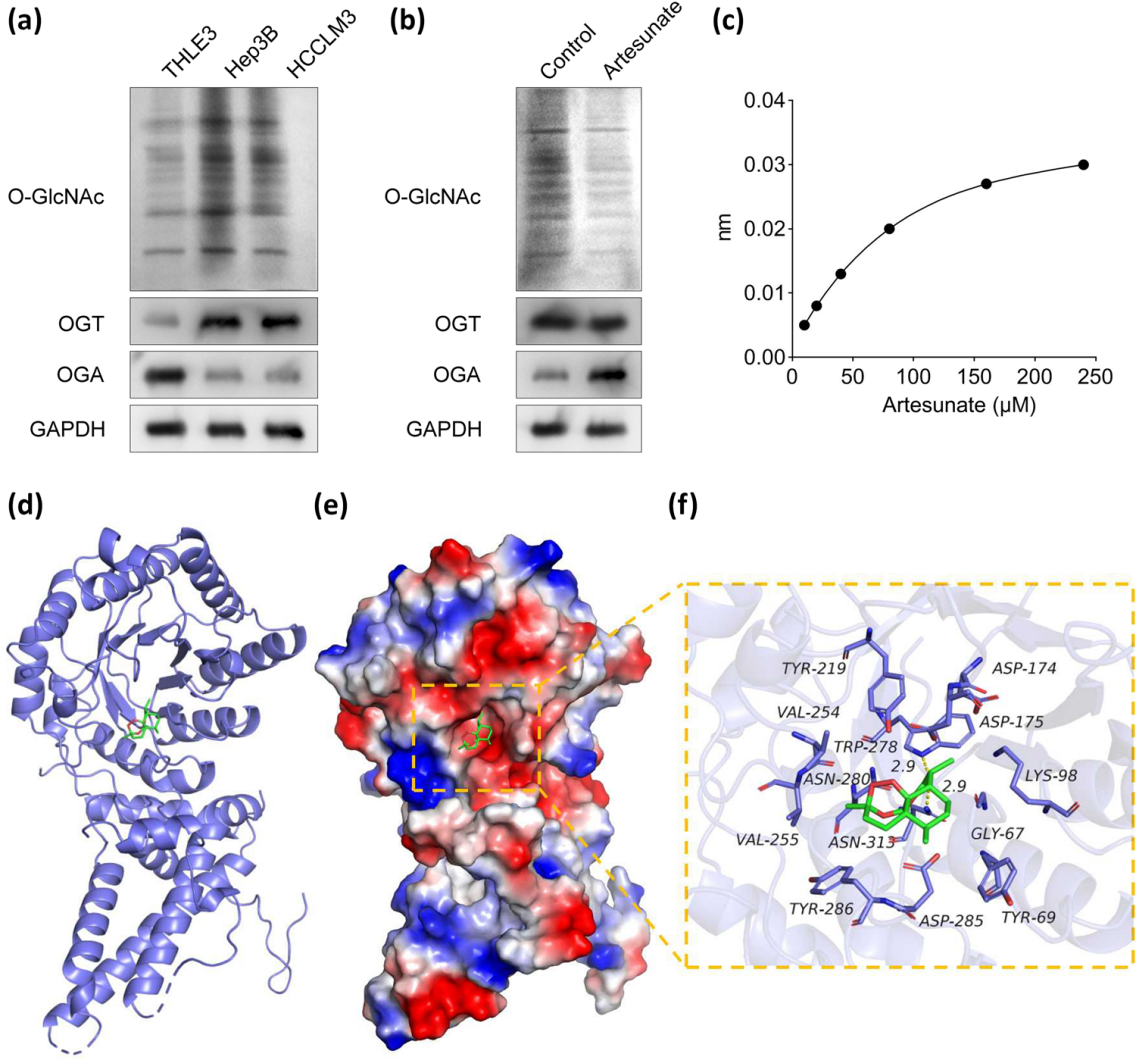
Artesunate inhibits OGA-mediated *O*-GlcNAcylation. (a) Immunoblotting was performed to detect the total *O*-GlcNAcylation level and protein levels of OGT and OGA. (b) The effect of artesunate on the total *O*-GlcNAcylation level and protein levels of OGT and OGA was evaluated using immunoblotting. (c) BLI analysis of the binding between artesunate and OGA. (d) The 3D structure of artesunate and the OGA binding complex was acquired using molecular docking. (e) The electrostatic surface of the protein OGA. (f) The detailed binding mode of artesunate and OGA.

*O*-GlcNAcylation has been implicated in the initiation, progression, and metastasis of HCC [22]. To elucidate the molecular mechanisms underlying the effects of artesunate, we investigated its impact on protein *O*-GlcNAcylation. As shown in Figure 3a, total *O*-GlcNAcylation levels were significantly elevated in Hep3B and HCCLM3 cells compared to THLE3 cells. OGT and OGA, the enzymes responsible for the addition and removal of *O*-GlcNAc modifications, respectively, were also examined. We observed that OGT levels were upregulated, while OGA levels were downregulated in HCC cells relative to THLE3 cells (Figure 3a). Further immunoblotting analysis revealed that artesunate treatment reduced total *O*-GlcNAcylation levels and increased OGA expression, but had no effect on OGT levels (Figure 3b). Biolayer interferometry (BLI) demonstrated strong binding between artesunate and OGA (Figure 3c), and molecular docking analysis further confirmed this interaction (Figure 3d–f). These findings collectively suggest that artesunate suppresses *O*-GlcNAcylation in HCC by directly binding to OGA.

### 3.4 Artesunate hampers HCC cell migration and invasion via increasing OGA expression

**Figure 4:**
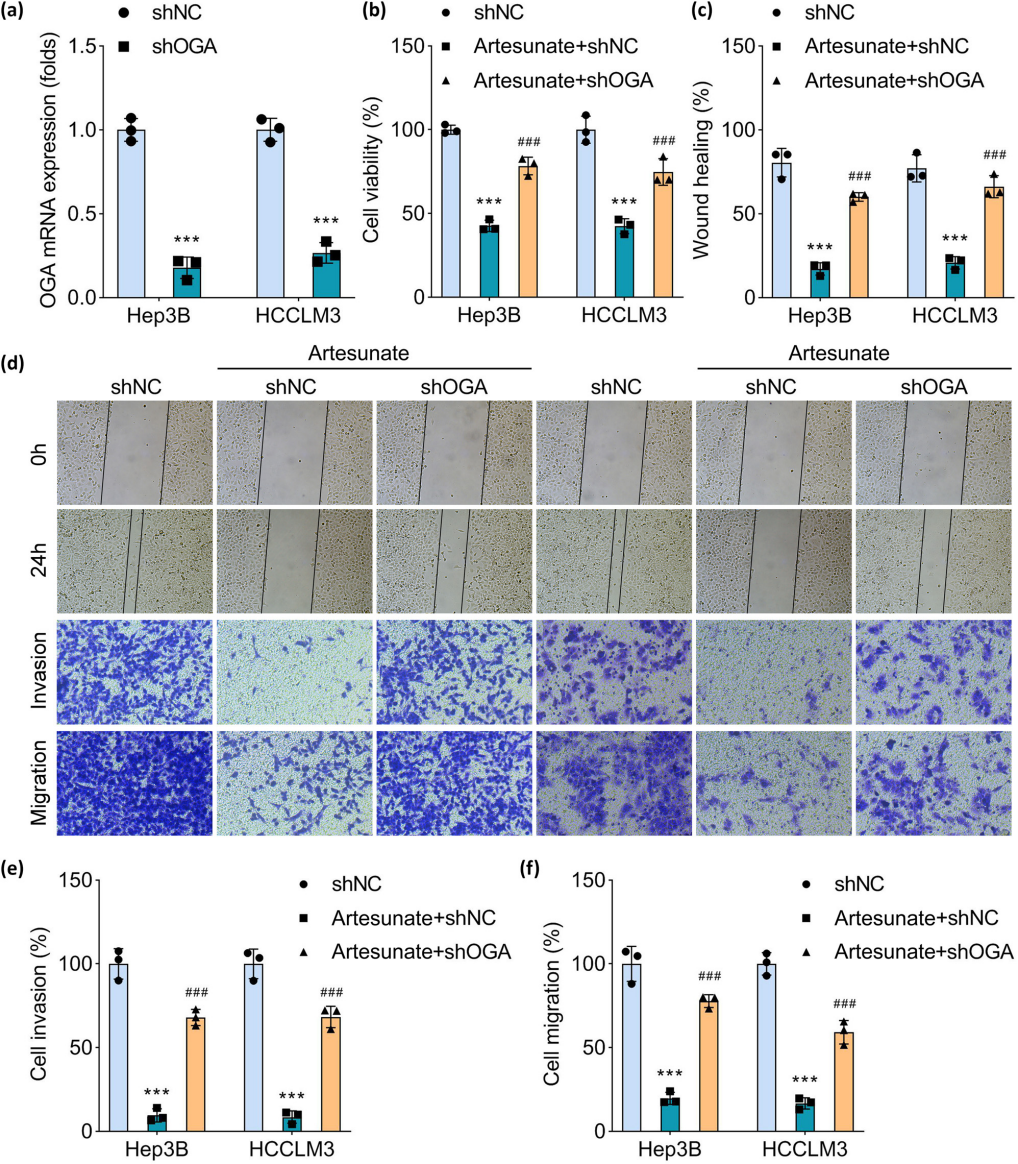
Artesunate hampers HCC cell migration and invasion via increasing OGA expression. (a) OGA mRNA expression was measured in Hep3B and HCCLM3 cells transfected with shNC and shOGA using qRT-PCR. (b) HCC cells were treated with artesunate and transfected with shOGA; cell viability was assessed using the cell counting kit-8. (c) Quantification results of the wound healing assay. (d) Represent images of wound healing and Transwell assays. (e) Cell invasion and (f) migration analyzed using the Transwell assay were quantified. ***P < 0.001 vs the shNC group. ^###^ *P* < 0.001 vs the artesunate + shNC group.

To determine whether OGA regulates HCC cellular behaviors, shOGA and shNC were transfected into Hep3B and HCCLM3 cells. OGA expression was significantly reduced in shOGA-transfected cells compared to shNC-transfected cells (Figure 4a). Artesunate treatment inhibited cell viability; however, this effect was attenuated by OGA knockdown (Figure 4b). Similarly, artesunate suppressed cell migration and invasion, but these inhibitory effects were reversed in OGA-depleted cells (Figure 4c–f). These results demonstrate that silencing OGA counteracts the artesunate-induced suppression of cell viability, migration, and invasion in HCC cells.

### 3.5 OGA inhibits *O*-GlcNAcylation of ZEB1

**Figure 5:**
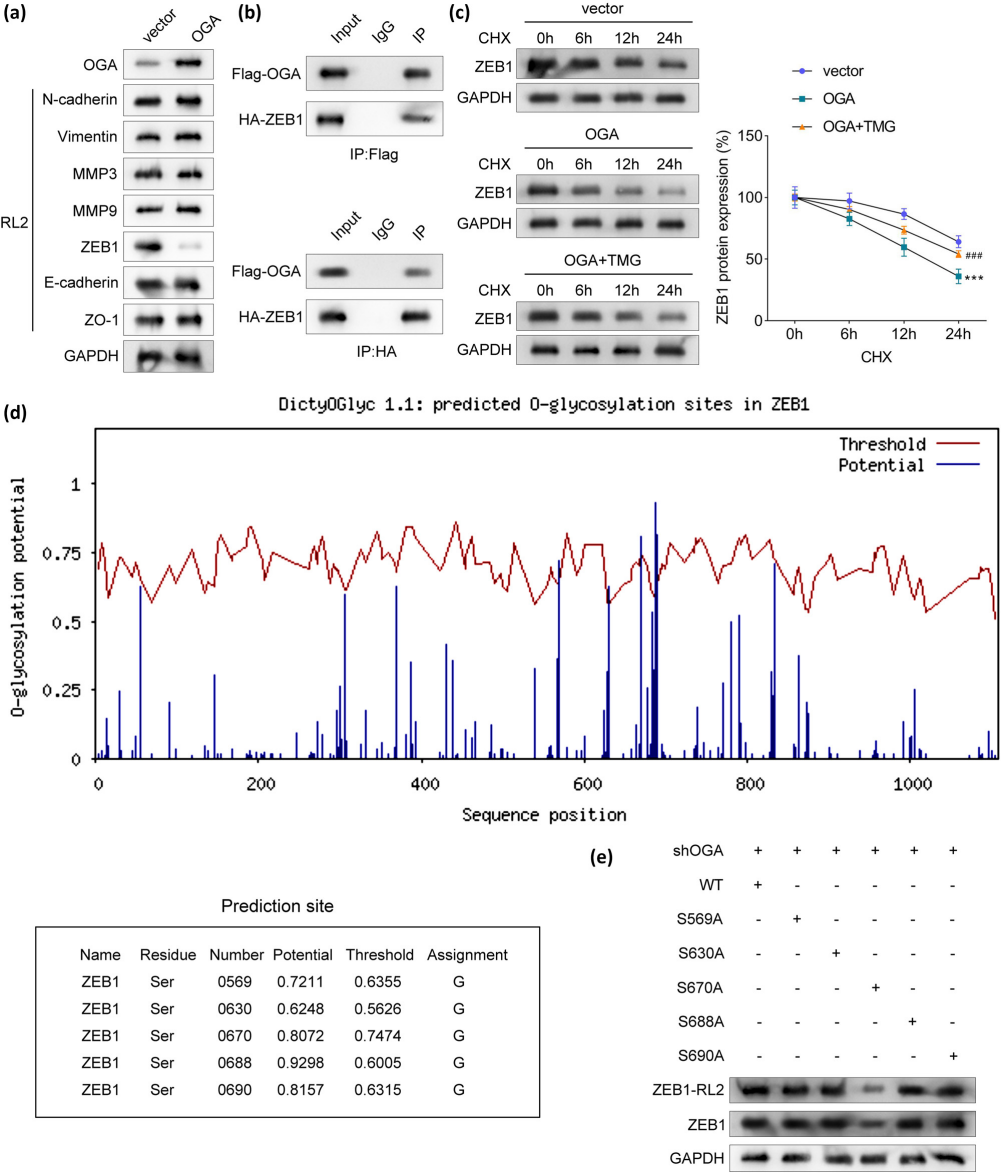
OGA inhibits *O*-GlcNAcylation of ZEB1. (a) The effect of OGA on the *O*-GlcNacylation levels of tumor metastasis-related proteins, including N-cadherin, Vimentin, MMP3, MMP9, ZEB1, E-cadherin, and ZO-1. (b) The interaction between OGA and ZEB1 was assessed using immunoprecipitation. (c) Effect of OGA-mediated *O*-GlcNAcylation on the stability of ZEB1 protein. (d) Potential *O*-GlcNAcylation sites in ZEB1 protein. (e) ZEB1 *O*-GlcNAcylation sites were analyzed using immunoprecipitation and immunoblotting. ***P < 0.001 vs the vector group. ^###^ *P* < 0.001 vs the OGA group.

OGA is known to remove *O*-GlcNAcylation modifications from proteins. To identify potential *O*-GlcNAc-modified proteins involved in tumor metastasis, we screened several metastasis-related proteins and assessed their *O*-GlcNAcylation levels. Among these proteins, overexpression of OGA specifically reduced the *O*-GlcNAcylation levels of ZEB1 (Figure 5a). Furthermore, co-immunoprecipitation assays confirmed that OGA physically interacts with ZEB1 (Figure 5b). Overexpression of OGA decreased ZEB1 protein stability, while inhibition of OGA restored ZEB1 stability (Figure 5c). Bioinformatic analysis predicted multiple potential *O*-GlcNAcylation sites in ZEB1 (Figure 5d). Among these, the five highest-probability sites were selected for further validation. Mutation of the S670 site significantly reduced *O*-GlcNAcylation levels and downregulated ZEB1 protein expression compared to WT-ZEB1 (Figure 5e), indicating that S670 is a critical *O*-GlcNAcylation site. These findings collectively demonstrate that OGA inhibits *O*-GlcNAcylation of ZEB1 at the S670 site, thereby reducing ZEB1 protein stability.

### 3.6 Knockdown of ZEB1 inhibits migration and invasion of HCC cells

**Figure 6:**
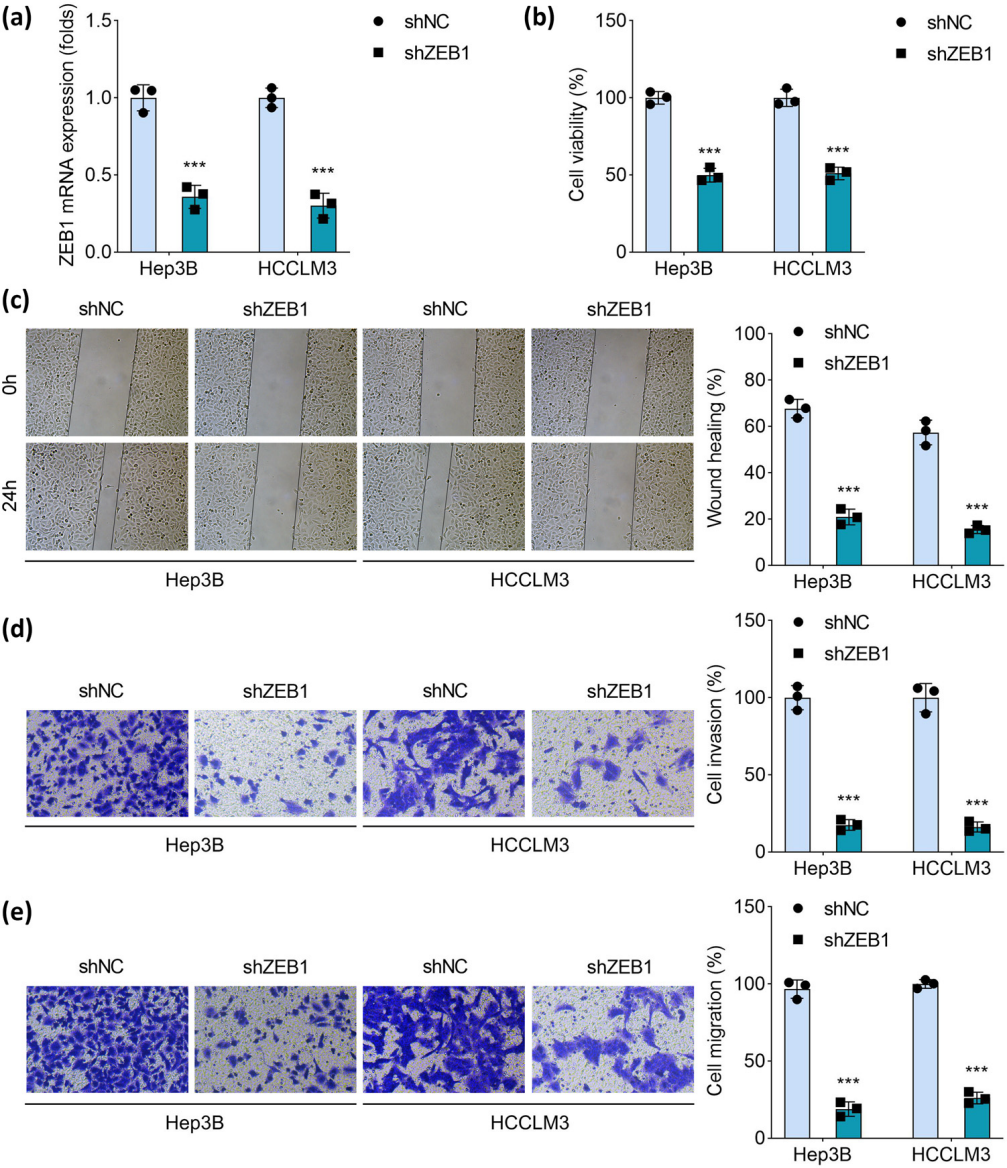
Knockdown of ZEB1 inhibits migration and invasion of HCC cells. (a) ZEB1 mRNA expression was measured in Hep3B and HCCLM3 cells transfected with shNC and shZEB1 using qRT-PCR. After transfection, (b) cell viability was assessed using the cell counting kit-8. (c) Cell migration was evaluated using the wound healing assay, and the percentage of wound healing was quantified. (d) Cell migration was analyzed using. (e) Cell invasion was evaluated using the Transwell assay and quantified. ***P < 0.001 vs the shNC group.

The effect of ZEB1 on cellular processes, we transfected shNC and shZEB1 into Hep3B and HCCLM3 cells. ZEB1 expression was downregulated after shZEB1 transfection, compared with shNC (Figure 6a). Next, we found that knockdown of ZEB1 inhibited HCC cell viability, migration, and invasion (Figure 6b–e). The findings suggest that ZEB1 acts as an oncogene in HCC.

## 4 Discussion

Artesunate, a well-established antimalarial drug, has garnered increasing attention for its anti-tumor properties. Numerous studies have explored its effects on various cancers, elucidating its mechanisms of action and molecular targets. For instance, artesunate inhibits colorectal cancer cell proliferation by inducing autophagy and disrupting mitochondrial function [23]. In head and neck cancer, artesunate promotes ferroptosis and enhances cisplatin sensitivity [24]. Similarly, in thyroid cancer, artesunate suppresses cell growth, metastasis, and induces apoptosis by inactivating the PI3K/AKT/FKHR pathway [25]. Artesunate has also emerged as a promising therapeutic candidate for HCC. Previous studies have demonstrated its ability to alleviate sorafenib resistance in HCC [15,16,26], potentially enhancing sorafenib efficacy and improving patient outcomes. The mechanisms underlying artesunate’s anti-cancer effects are multifaceted. Chen et al. [27] reported that artesunate induces autophagy by targeting glucosylceramidase, while Jiang et al. [28] highlighted its role in promoting ferroptosis by disrupting iron homeostasis in HCC cells. However, the impact of artesunate on HCC metastasis remains poorly understood. In this study, we demonstrated that artesunate significantly inhibits HCC cell viability, migration, and invasion, suggesting its potential as a therapeutic agent for metastatic HCC. However, we only performed *in vitro* experiments. Before a preclinical study, an animal study is needed to evaluate the effect of artesunate on tumor growth and metastasis.

Given the critical role of *O*-GlcNAcylation in cancer progression, we investigated whether artesunate modulates this post-translational modification to exert its anti-tumor effects. Our results revealed that *O*-GlcNAcylation levels were elevated in HCC cells and significantly reduced following artesunate treatment. Concurrently, artesunate upregulated OGA expression, suggesting that OGA-mediated *O*-GlcNAcylation plays a key role in the anti-HCC effects of artesunate. OGA is known to be dysregulated in various cancers and is implicated in tumor progression, metastasis, and immune modulation [29]. OGA has been reported to function as a tumor suppressor [30,31], including in HCC. Specifically, downregulation of OGA, mediated by RANBP2, has been shown to enhance *O*-GlcNAcylation levels, thereby promoting HCC cell viability and invasion [32]. Additionally, astragalus, an active component of Astragalus membranaceus, has been reported to induce HCC cell apoptosis and endoplasmic reticulum stress by upregulating OGA expression [33]. In this study, we demonstrated that artesunate directly binds to OGA and that OGA knockdown reverses the inhibitory effects of artesunate on HCC cell viability, migration, and invasion. These findings collectively indicate that artesunate suppresses HCC progression by upregulating OGA expression and modulating *O*-GlcNAcylation.

OGA regulates tumor progression by inhibiting the *O*-GlcNAcylation of substrate proteins. In this study, we identified that ZEB1 undergoes *O*-GlcNAcylation. As a transcription factor implicated in EMT, ZEB1’s overexpression serves as a predictor of poor survival outcomes in various cancers [34]. Noteworthily, ZEB1 modulates tumor metastasis through its influence on tumor cell motility and dissemination [35]. Extensive research has elucidated the role of ZEB1 in HCC, demonstrating that it acts as a tumor promoter by facilitating metastasis, the Warburg effect, chemoresistance, and impeding tumor cell apoptosis [36,37,38]. Additionally, *O*-GlcNAcylation of ZEB1 has been shown to promote lipid peroxidation and ferroptosis in pancreatic cancer cells [39]. However, whether ZEB1 is subject to *O*-GlcNAcylation in HCC remains unclear. *O*-GlcNAcylation has been reported to contribute to protein structure and functions, including localization, stability, and chromatin remodeling [40]. Our findings reveal that OGA specifically inhibits *O*-GlcNAcylation of ZEB1 at the S670 site, thereby promoting its protein degradation, suggesting that *O*-GlcNAcylation is involved in modulating ZEB1 protein stability, consistent with a previous study [39]. These findings will provide new insights into the role of post-translational modifications of proteins and the regulatory mechanism of ZEB1 in HCC. However, we speculated that *O*-GlcNAcylation may directly affect ZEB1 protein conformation, either through competitive interaction with other post-translational modifications such as phosphorylation or through the ubiquitin-proteasome pathway affecting ZEB1stability, which will be further studied in our work.

In conclusion, this study demonstrates that artesunate inhibits cell migration and invasion in HCC by enhancing OGA expression, which in turn suppresses the *O*-GlcNAcylation of ZEB1. These findings suggest that artesunate may represent an effective therapeutic agent for the treatment of HCC and offer novel insights into its mechanism of action.

## Supplementary Material

Supplementary Figure
