# Supplementary figures and images for: Artesunate inhibits hepatocellular carcinoma cell migration and invasion through OGA-mediated O-GlcNAcylation of ZEB1
Source: Open Life Sci. 2025 Aug 5;20(1):20251109. doi: 10.1515/biol-2025-1109 (PMC12326304; doi:10.1515/biol-2025-1109)

# Supplementary material

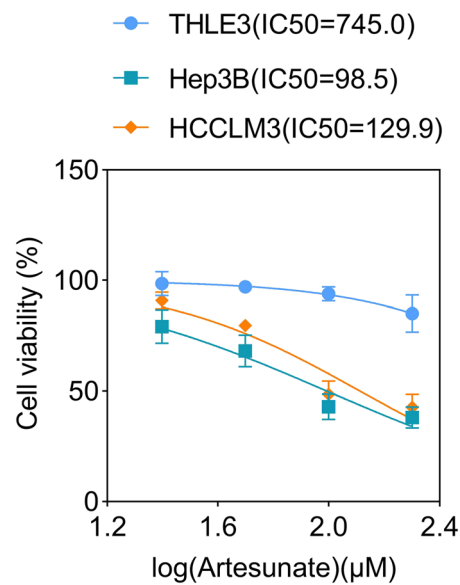

**Figure S1:** IC50 of artesunate according to cell viability assay.

Supplement: Supplementary Figure [file biol-2025-1109-sm.pdf]
